# Bragg Spot Finder (BSF): a new machine-learning-aided approach to deal with spot finding for rapidly filtering diffraction pattern images

**DOI:** 10.1107/S1600576724002450

**Published:** 2024-04-26

**Authors:** Jianxiang Dong, Zhaozheng Yin, Dale Kreitler, Herbert J. Bernstein, Jean Jakoncic

**Affiliations:** a Department of Computer Science, College of Engineering and Applied Sciences, Stony Brook University, Upton, NY, USA; b National Synchrotron Light Source II, Brookhaven National Laboratory, Building 745, Upton, NY, USA; c Ronin Institute for Independent Scholarship, c/o NSLS-II, Brookhaven National Laboratory, Building 745, Upton, NY, USA; Uppsala University, Sweden; The European Extreme Light Infrastucture, Czechia

**Keywords:** Bragg Spot Finder, BSF, Bragg Spot Detection, BSD, Bragg reflections, weak intensities, machine learning, filtering, X-ray diffraction, microbeams, diffraction spot search, real time processing

## Abstract

Macromolecular crystallography contributes significantly to understanding diseases and, more importantly, how to treat them by providing atomic resolution 3D structures of proteins. This is achieved by collecting X-ray diffraction images of protein crystals from important biological pathways. Spotfinders are used to detect the presence of crystals with usable data, and the spots from such crystals are the primary data used to solve the relevant structures. Having fast and accurate spot finding is essential, but recent advances in synchrotron beamlines used to generate X-ray diffraction images have brought us to the limits of what the best existing spotfinders can do. This bottleneck must be removed so spotfinder software can keep pace with the X-ray beamline hardware improvements and be able to see the weak or diffuse spots required to solve the most challenging problems encountered when working with diffraction images. In this paper, we first present Bragg Spot Detection (BSD), a large benchmark Bragg spot image dataset that contains 304 images with more than 66 000 spots. We then discuss the open source extensible U-Net-based spotfinder Bragg Spot Finder (BSF), with image pre-processing, a U-Net segmentation backbone, and post-processing that includes artifact removal and watershed segmentation. Finally, we perform experiments on the BSD benchmark and obtain results that are (in terms of accuracy) comparable to or better than those obtained with two popular spotfinder software packages (*Dozor* and *DIALS*), demonstrating that this is an appropriate framework to support future extensions and improvements.

## Abstract 2

Bragg Spot Finder (BSF) is a U-Net-based spotfinder with image preprocessing, a U-Net segmentation backbone, and post-processing that includes artifact removal and watershed segmentation. BSF is supported by the Bragg Spot Detection (BSD) benchmark image dataset containing more than 300 images with more than 66 000 spots.

## 1. Introduction

Macromolecular crystallography (MX) remains the primary method used to determine high-resolution 3D structures of proteins. Drug design projects rely mostly on crystallography because it is an unmatched fast and reliable method for well studied systems. In order to determine the 3D crystal structure of a protein that is expressed and purified at the desired purity and concentration, one has to crystallize the sample (Yin *et al.*, 2014 ▸; Teplitsky *et al.*, 2015 ▸; Nam, 2023 ▸; Henkel *et al.*, 2023 ▸), collect diffraction data from the crystal, reduce the data, and solve the structure using either experimental phasing methods or the molecular replacement method.

The complexity of structural biology projects is increasing, and this often results in the use of more challenging crystals from which to collect diffraction data. This means that more crystals have to be tested before the best one(s) are used for data collection. Many crystallographic studies require several visits to synchrotron facilities for data collection to ensure the best outcome. ‘Best’ means sufficient data for a structure solution at the desired resolution with corresponding electron density maps displaying features supporting conclusive results.

This is where fast spotfinders are employed, when searching for crystals of best diffraction volumes from a large crystal in what is called rastering experiments. In a rastering experiment (grid scan), the sample is translated and X-ray diffraction data are collected at every grid point. The diffraction patterns are analyzed by the fast spotfinder application and a heat map is computed and displayed. Similarly to a cartographic heat map, the intensity of the colored heat map is proportional to a factor that has been selected by the end user: the number of reflections, the total intensity, the resolution or a scoring factor. Users select the best area of the sample for data collection by reviewing diffraction data for each point of the map with a strong signal. This process is automated for automated workflows. The sample is then centered and data collection for structural analysis is executed; complete datasets are analyzed using several available data reduction programs and workflows.

All data reduction programs used for processing complete datasets made of contiguous diffraction images contain a spotfinder. The spotfinder is used initially to locate Bragg reflections for pattern indexing. This is the step where the geometry of the experiment and the location of the reflections on the detector plane are used to estimate the crystal symmetry and the cell parameters. Indexing is then used to predict more reflections and cycles of refinement are used to refine as many parameters as possible according to the positions of all found reflections.

Spot finding is based on the approaches established when film was digitized with drum scanners (Kabsch, 1977 ▸). Although experimental collection for structural analysis might still be achieved from a scan of a single large crystal, our concern is primarily with the more challenging cases of small crystals or the use of microbeams to probe larger non-uniform crystals. The software for spot finding in current use derives from *XDS* (Kabsch, 2010 ▸), *LABELIT*, *DISTL*, *cctbx.spotfinder* and *DIALS* spotfinder (Sauter *et al.*, 2004 ▸, 2013 ▸; Zhang *et al.*, 2006 ▸; Parkhurst *et al.*, 2015 ▸), *Dozor* (Melnikov *et al.*, 2018 ▸), *HKL-2000* (Minor *et al.*, 2002 ▸), *CrystFEL* (White *et al.*, 2012 ▸), and other packages. For more on the complex history of X-ray crystallography including spot finding, see Harry Powell’s 2017 review (Powell, 2017 ▸).

However, in all of these cases, focusing on spot finding prior to integration, real time feedback was not a required built-in feature. Additionally, a minimum number of reflections is required for successful indexing.

In a rastering experiment, one tries to minimize the energy dose deposited on the sample and maximize the potential number of reflections by rotating the sample by a minute amount (50 to 200 millidegrees is the typical range). However, we found that collecting stills – where the crystal is not rotated, only translated – gives a better chance for optimal crystal locations for crystals smaller than the footprint of the beam (10 µm or less). This results in a lower number of reflections meeting the Bragg condition or in only partial or weak reflections. The weakest reflections are those with a minimum of one count in each peak pixel above the local background. Since the purpose of rastering is to find the crystal(s), it is also often the case that very few reflections are observed.

In short, the ideal spotfinder should be fast enough to keep up with the fastest data collection rate (about 1000 FPS for a 4–15 megapixel area detector) while being sensitive enough to detect a single low-resolution weak reflection. Since the idea is not to overexpose the samples, one can imagine a workflow where, if the initial grid scan returned an empty map, one could repeat that grid scan with a significantly higher transmitted flux.

Currently, two existing applications are in particularly heavy use at synchrotron beamlines, *dials.find_spots_server/_client* (*DIALS*) (Zhang *et al.*, 2006 ▸; Winter *et al.*, 2018 ▸; Sauter, 2011 ▸) and *Dozor* (Melnikov *et al.*, 2018 ▸). Many factors impact the actions necessary to optimize or tune the parameters of any spot finding algorithm. First and foremost are the characteristics of the X-ray source and of the X-ray detector. In this case, the data were collected at AMX – the highly automated macromolecular crystallography (17-ID-1) beamline at National Synchrotron Light Source II (Schneider *et al.*, 2022 ▸). Our benchmarking at AMX indicated that *DIALS* provides false positives as well as undercounted reflections (*DIALS* does not offer optimal detection of Bragg reflections). When ice rings are present, ice ring filtering may require a second software pass which further increases delays. Ideally, an optimal Bragg spot finder would not require manual tuning of parameters such as the number of pixels in a spot, *i.e.* the minimum pixel count. We also found that *Dozor*, though it can keep up with real time feedback, at times fails to detect weak reflections (Bragg reflections very close to the local average background) and in such cases returns an empty heat map.

Some of the necessary spot finding parameter settings can be derived from the known average characteristics of the relevant beamline in advance (such as background related to sample environment). Some can be determined dynamically during one or more data collection passes, and some are determined after the fact by careful manual or algorithmic examination of experimental datasets. One of the main reasons to use AI techniques is that it can make important tuning decisions on the fly.

Here we are exploring the potential exploitation of a machine-learning (ML) or artificial intelligence (AI) application to overcome some of the limitations of *DIALS* and *Dozor*. The value of AI for spot finding with a reference dataset was established by Ke *et al.* (2018 ▸) in an XFEL context. This reference dataset has also been used by Rahmani *et al.* (2023 ▸) and Nawaz *et al.* (2023 ▸). Our focus is on reliability and speed in a synchrotron context for optimal crystal detection using rastering, which differs from the XFEL context. It is important to make the new spotfinder faster so it can keep up with new upcoming detectors at 2000 FPS for a 15 MP detector. Integrating detectors with significantly improved framing rates (Grimes *et al.*, 2023 ▸) will require dedicated hardware solutions based on field-programmable gate arrays (FPGAs).

In the following we discuss the spotfinder Bragg Spot Finder (BSF), which in its present form is based on U-Net, a convolutional neural network for biomedical image segmentation (Ronneberger *et al.*, 2015 ▸). BSF is supported by the Bragg Spot Detection (BSD) benchmark image dataset containing more than 300 images with more than 66 000 spots.

## 2. Collecting the BSD dataset

In this section, we outline the BSF data collection pipeline, including image capturing and annotation generation, and list the statistics of the BSD dataset.

### 2.1. Image capturing

AI/ML applications need large training sets that represent a vast array of data that have been observed. This is to develop an unbiased application trained on all possible data. This is an extremely difficult goal to achieve. AI/ML software development needs training data for which the expected results have been clearly established – what we call the ground truth. To provide the training data needed, we used real diffraction data from AMX. The primary scientific mission of AMX is to support routine structure determination for the most challenging projects using automated data collection for all samples. The beam size is 7 × 5 µm, the photon flux is 4.5 × 10^12^ photons s^−1^ and the area detector is a Dectris Eiger X 9M, running at 200 Hz for most rastering experiments. These beam parameters are amongst the best worldwide and allow the study of weakly diffracting crystals, measuring 5 µm in the longest dimension. As a result, we have access to a very broad range of diffraction frames in terms of qualitative and quantitative parameters, and a very broad range of samples yielding a wide variety of diffraction patterns. The average oscillation width per frame for rastering and for data collection is 0.1°, and the common range in use is 0.05–0.2°. AMX staff flagged diffraction patterns deemed useful for building up the two datasets used to develop the BSF application to represent the broadest possible data: the training set and the testing set. Diffraction data with the following features were flagged and saved: diffraction with ice rings or strong ice rings; diffraction to very high resolution; diffraction from very large cell parameters, weakly diffracting samples or multiple lattices; diffraction patterns with no protein crystal diffraction; anisotropic diffraction; diffraction from samples with very high mosaicity, elongated spots, large spots or very low mosaicity; diffraction from uniform crystals displaying sharp reflections; and, when possible, combinations of these features. The diffraction frames from rastering experiments and from data collection experiments were saved. A total of 304 diffraction frames were used for this study.

### 2.2. Annotation generation

Once the frames are selected, they go through a semi-automated process that executes the two most commonly used applications, *dials.find_spots* and *Dozor*, using default and/or optimized parameters. A duplicate from the *Dozor* application with an output file that can be read by the *ADXV* (Arvai, 2021 ▸) application is also generated. *ADXV* is a diffraction image viewer that can also open a spot file for manual inspection, including manual addition and removal of reflections. This was used for all training set diffraction images to generate a ground truth. The ground truth was generated by expert crystallographers with a collective experience of 34 years. Each inspected about half of the total number of diffraction frames to be analyzed. They used *ADXV* to manually inspect the frames and remove or add Bragg reflections, adjusting the image contrast so that the best contrast was used to generate the best Bragg reflection assignments on all the frames (304 in total: 245 for training and 59 for testing). It took on average 5 min per frame for the manual annotation and was performed locally to maximize image quality as opposed to relying on remote connection applications that typically compress video feeds.

### 2.3. Overview of the BSD dataset

The BSD dataset contains 304 high-resolution (3269 × 3110) images with manual annotations (spot point coordinate positions) and software (*Dozor* and *DIALS*) predictions. We split off 20% of the images for the evaluation, leading to 245 and 59 for training and test sets, respectively. Table 1 ▸ summarizes some statistics of the BSD dataset.

## 3. Methods

### 3.1. Problem formulation

**Figure 1:**
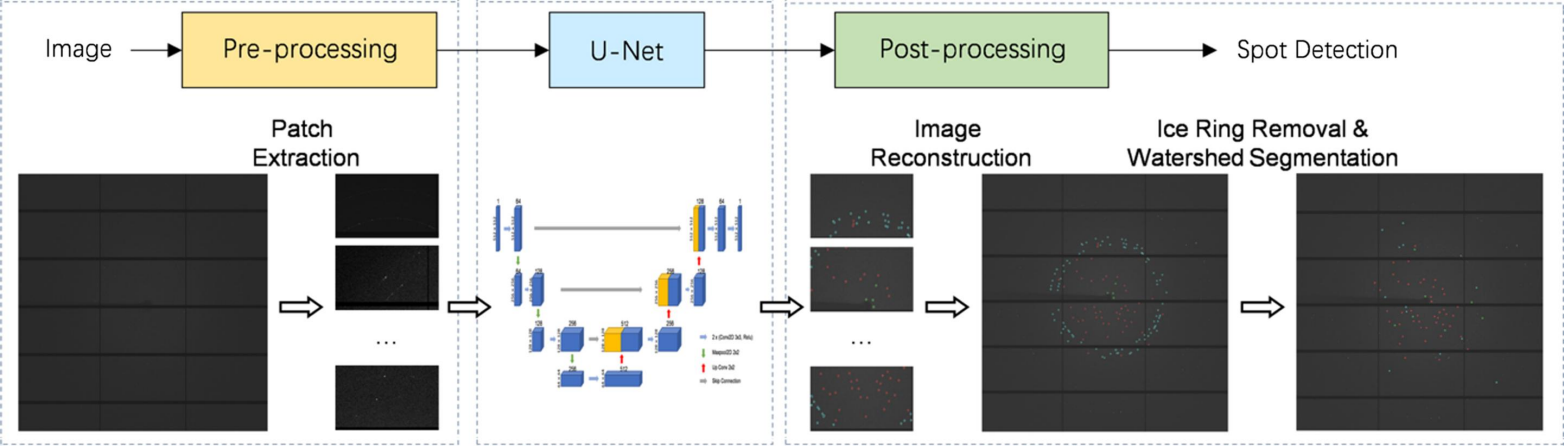
Overview of the BSF method.

We formulate the spot detection problem as an image segmentation task and the goal is to segment an image into two categories, namely foreground spots and background. Fig. 1 ▸ shows the pipeline of BSF, which includes data pre-processing, U-Net for image segmentation and post-processing.

### 3.2. Data pre-processing

The dataset is stored in .cbf files, so first we convert them into grayscale images. Because of memory issues and the size of the high-resolution (3269 × 3110) images in the dataset, we cut images into patches of 512 × 512 before feeding them into the model, which performs patch-level spot segmentation. The ground truth annotations of the BSD dataset are the spot point positions. For the segmentation task, we need to generate mask annotations. Specifically, we generate a diamond-shaped mask with a radius of 10 pixels centered at the ground truth spot position.

### 3.3. U-Net for image segmentation

**Figure 2:**
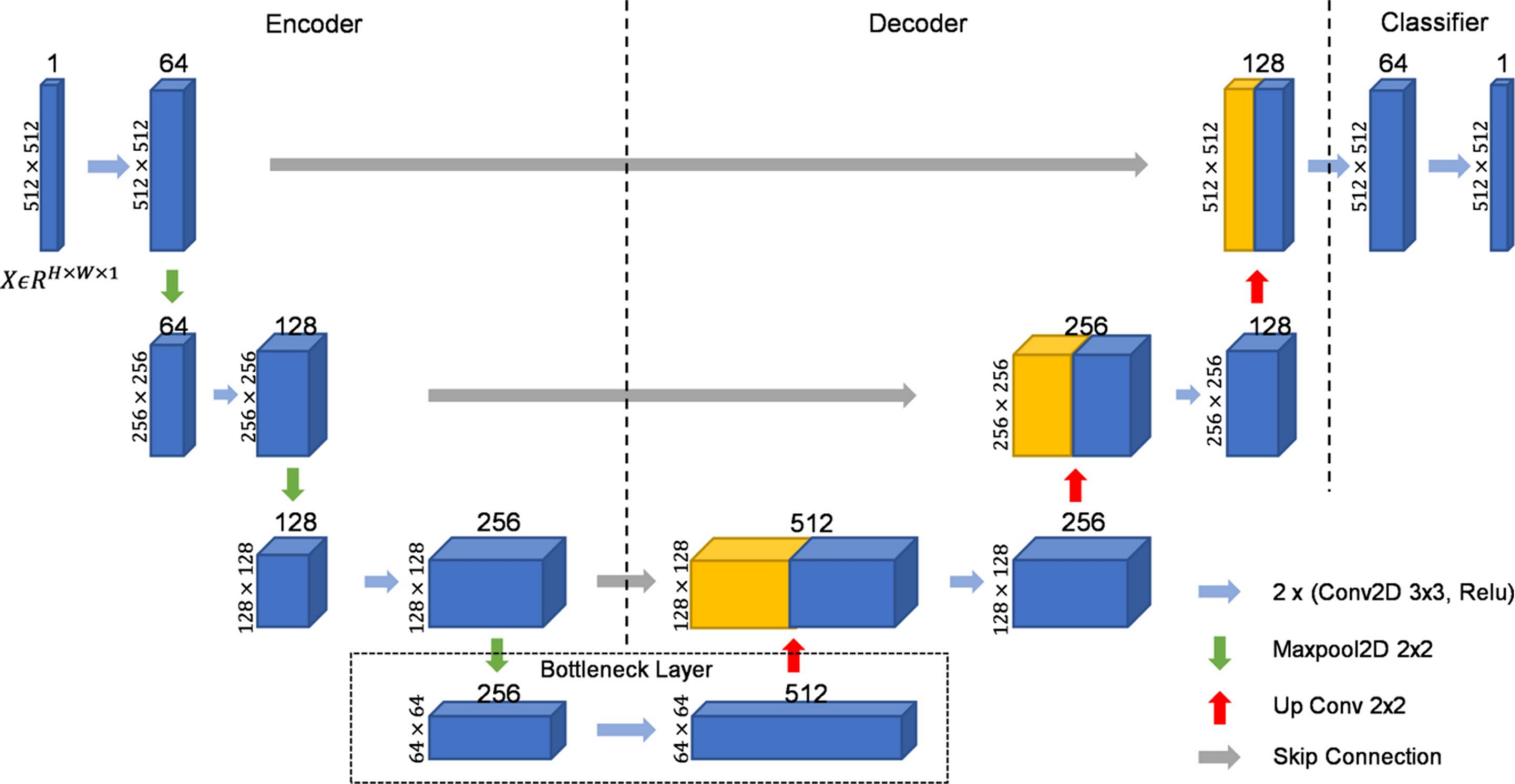
Our U-shaped model architecture for image segmentation which consists of a set of convolutions, up/down sampling and a fully connected classifier, as detailed in Section 3.3.

In this section, we present an overview of our backbone model performing spot segmentation on Bragg spot images. We adopted U-Net (Ronneberger *et al.*, 2015 ▸) as the backbone and the details of our U-shaped model architecture are shown in Fig. 2 ▸.

As shown in Fig. 2 ▸, the input image patch 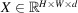 (*H* = *W* = 512 are the width and height of the image patch, and the initial feature dimension *d* = 1) is fed into the encoder. It first performs convolution operations that increase the feature dimension of *X* from 1 to 64. The output is then passed to a pooling layer that performs a down-sampling process to decrease the size by 2, leading to a new 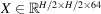 . The encoder will repeat the convolution and pooling operations three times and obtain the feature map 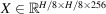 in the bottleneck layer. The bottleneck layer has another convolution layer to further increase its feature dimensions from 256 to 512. Then it will be passed to the decoder which employs up-sampling to increase its size by 2 and convolution operations to reduce its feature dimensions. The output of the decoder will be 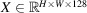 , which is the same size as the input image patch but with 128 feature dimensions. Afterwards, a classifier that consists of a fully connected layer with a rectified linear unit activation (Nair & Hinton, 2010 ▸) is adopted to generate the final prediction map 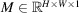 . Finally, the prediction map is mapped into a probabilistic classification map 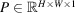 using a sigmoid function:

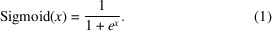

Each value in the classification map *P* represents its probability of being part of foreground spots. The overall model can be expressed as

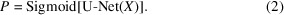

We train the U-Net model using focal loss (Lin *et al.*, 2017 ▸),

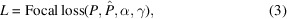

where *P* is the estimated probability mask of the model for the foreground spots, 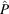 is the ground truth label, and α and γ are tunable weights to handle class imbalance and hard sample issues.

### 3.4. Post-processing

**Figure 3:**
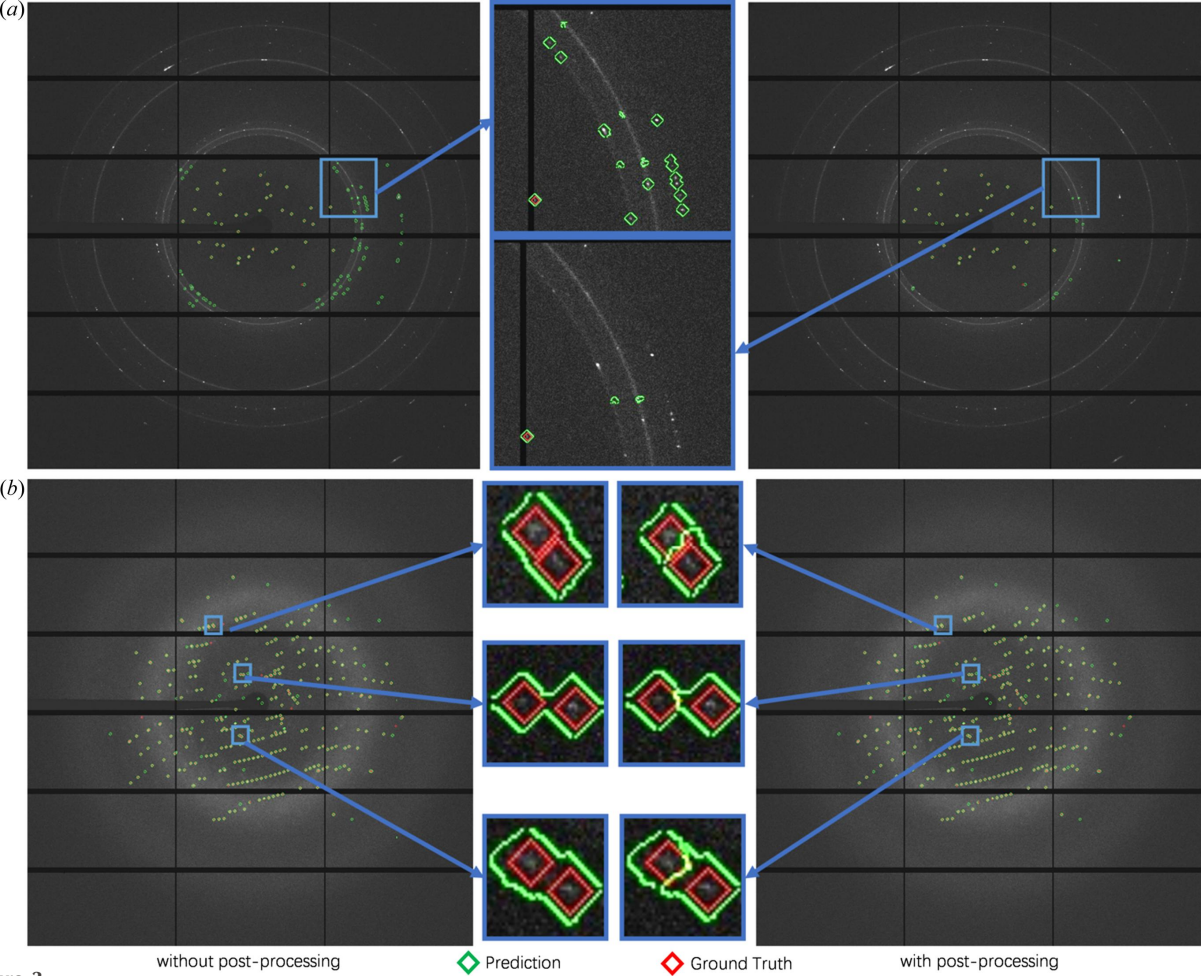
Our BSF spot predictions with and without any post-processing. Without post-processing, we can observe that the model (*a*) predicts some artifacts around the ice ring as spots (in green) and (*b*) detects a large region with multiple spots as a single spot (in green).

The outputs from the U-Net model cannot be directly used as the final predictions since it only makes patch-level segmentation predictions. In addition, some original inputs include artifacts arising from a foreign origin (ice rings, small molecules) generating diffracted intensities. For example, we can observe from Fig. 3 ▸(*a*) that there are some artifacts in the ice ring regions (elongated and twisted reflections) and the U-Net predictions will have a large number of false-positive predictions (predicting artifacts as spots) around ice ring regions. Moreover, if spots are densely distributed (small distance between spots), the model will predict a large spot instead of distinguishing them [shown in Fig. 3 ▸(*b*)]. Therefore, we designed a three-step post-processing to generate the final spot detection results based on the U-Net outputs.

(1) We reconstruct the whole image from all 512 × 512 pixel patches by stitching the patches to match their positions in the original image.

(2) For spots whose area is larger than the threshold τ_area1_, we adopt a watershed algorithm (Kornilov & Safonov, 2018 ▸) to split predicted large spots into smaller sub-spots and then mask out the predicted spots whose area is smaller than τ_area2_.

(3) We remove the predicted spots whose centers are located within a small distance of each ice ring (explained in Section 3.5).

We can observe the effectiveness of our post-processing from Fig. 3 ▸, where we split large-spot predictions into multiple smaller ones and remove false positives in the ice ring regions.

### 3.5. Ice ring spot removal

We perform post-processing to remove detected spots within known ice rings. Specifically, we calculate the ice ring positions on the basis of the experimental geometry (known resolutions, rotation axis, sample-to-detector distance, detector specifications and X-ray beam parameters of the ice rings). The ice ring center is recorded and its ‘radius’ in the image can be calculated as follows: 

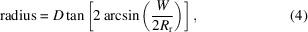

where *D* is the detector distance to the sample, *W* is the X-ray wavelength, and *R*_r_ is a known and fixed ice ring resolution. After obtaining all ice ring positions, we mask out the predicted spots whose distance in pixels to the nearest ice ring is smaller than a pre-defined threshold τ_ring_. In addition, we remove the predicted spots whose distance to the beam center is smaller than a threshold τ_beam_.

**Figure 4:**
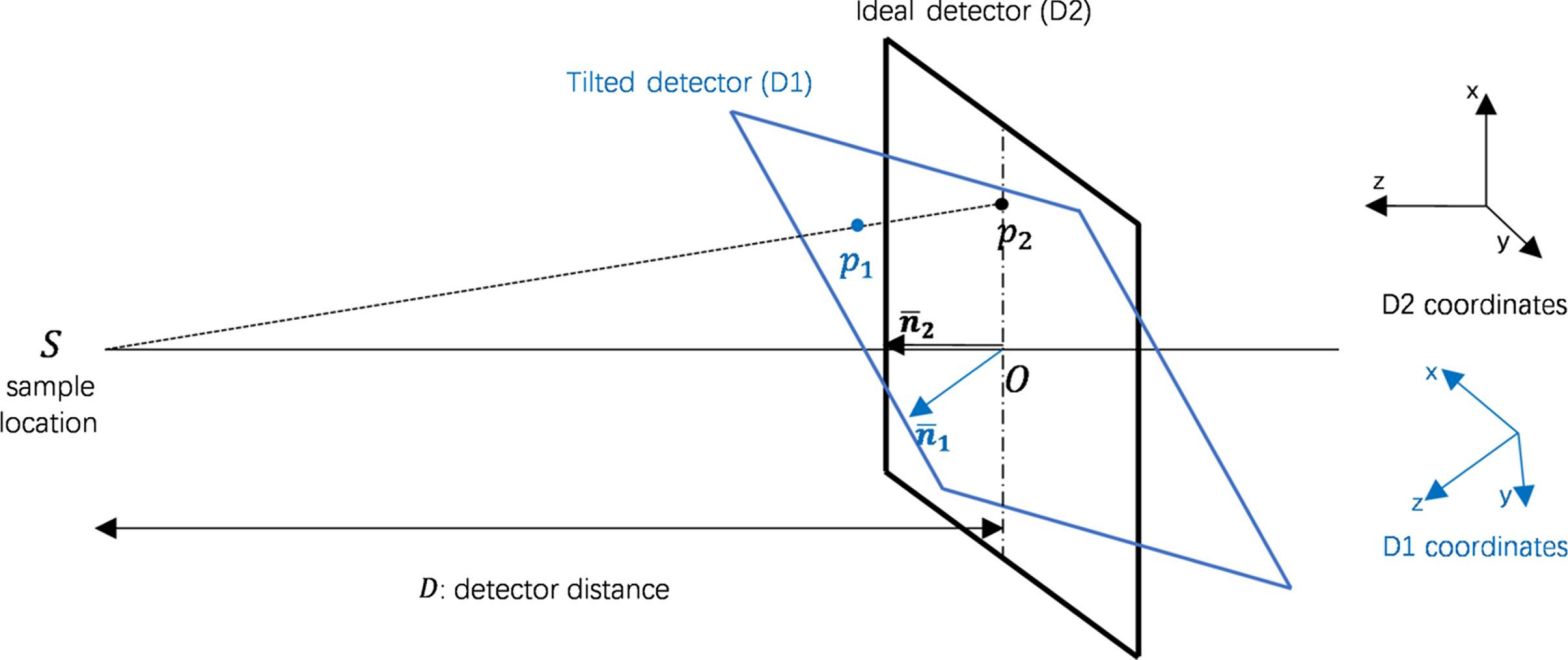
Illustration of the tilted detector and the ideal detector.

In practice, the detector orientation is not perfectly perpendicular to the incoming beam and has a tilting angle, as shown in Fig. 4 ▸. This will cause an ice ring distortion effect so the ice ring will be an ellipse rather than a circle. As a result, the ice ring spot removal will remove the true positives while leaving the false positives unchanged. Therefore, we perform a tilted detector calibration on the image based on the detector orientation.

Assuming we have a point **p**_0_ = (*x*_0_, *y*_0_) in the image plane of the tilted detector (*D*_1_), we first transform the point from the 2D image space of *D*_1_ to the point **p**_1_ = (*x*_1_, *y*_1_, *z*_1_) in the 3D space of *D*_1_ (*z* = 0 in the *D*_1_ coordinate system). Given the ideal detector (*D*_2_) orientation 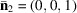 and tilted detector (*D*_1_) orientation 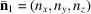 , we can calculate the rotation matrix based on the Rodrigues rotation formula (Galois, 1846 ▸): 

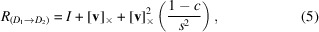




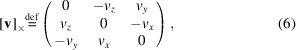

where 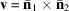 , *s* = ||**v**|| and 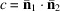 . Then, we apply the rotation transformation on the point **p**_1_ and obtain the point **p**_2_ in the 3D space of the ideal detector (*D*_2_): 

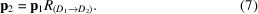

Finally, we apply the perspective projection on **p**_2_ = (*x*_2_, *y*_2_, *z*_2_) to obtain the point **p**_3_ = (*x*_3_, *y*_3_) in the 2D image plane of the tilted detector: 

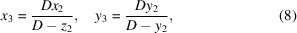

where *D* is the detector distance to the sample. We now have the mapping between a point **p**_0_ on the tilted detector and the corresponding point **p**_3_ on the ideal detector; we can either transform the spot position or distort the ice ring position, and then perform the ice ring spot removal.

**Figure 5:**
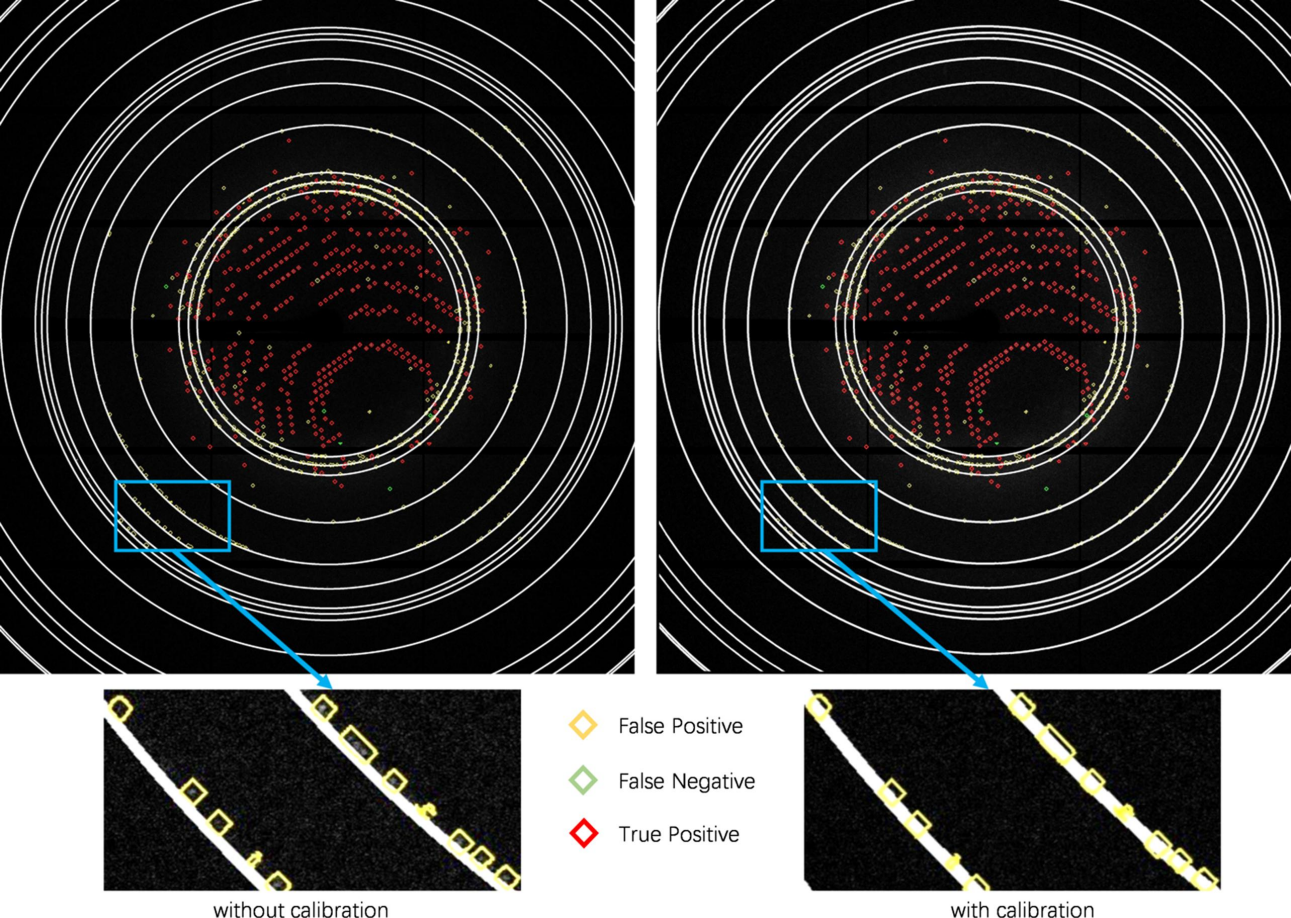
Effect of tilted detector calibrations on images for false-positive spot removal. The ice rings obtained with the tilted detector calibration are more accurate.

In Fig. 5 ▸, we show the calibration results and observe that the calibrated new ice rings correspond better to the calculated ice rings and are now closer to the false-positive predictions.

## 4. Experiment

We performed an experimental assessment on the BSD dataset and compared our results with the predictions of two commonly used software packages: *Dozor* and *DIALS*.

### 4.1. Evaluation metrics

We adopt the recall rate (Rec), precision rate (Pre) and F1 score to evaluate our model:

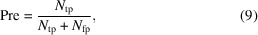




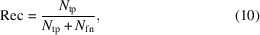




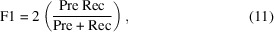

where *N*_tp_ is the number of correctly detected spots, *N*_fp_ is the number of incorrectly detected spots and *N*_fn_ is the number of missed spots. We regard the predicted spot as a correct prediction if the distance between the center of a predicted spot and the ground truth annotation position is less than the threshold *r* = 10 pixels.

### 4.2. Implementation details

In the experiment, we set the number of hidden units in different layers of U-Net to 64, 128 and 256. The thresholds for post-processing τ_ring_, τ_beam_, τ_area1_ and τ_area2_ were set at 3, 60, 400 and 60, respectively. The direction vector of the tilted detector is 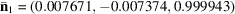 . The batch size was set to 8 and we trained our model using the Adam optimizer (Kingma & Ba, 2014 ▸) with a learning rate of 1 × 10^−3^. It takes about 3 h using a single Tesla V100 16 GB GPU to train the model.

### 4.3. Results and comparisons

We compared our BSF model with two commonly used software packages, namely *Dozor* and *DIALS*, as shown in Table 2 ▸. The image-level metrics are based on *N*_tp_, *N*_fp_ and *N*_fn_ for each image and then we take the average over all images, while the spot-level metrics are based on *N*_tp_, *N*_fp_ and *N*_fn_ of all the test set data. In the spot-level comparison, our model results in significantly higher performance compared with *DIALS* on all metrics, and we observe a higher F1 score and recall rate compared with *Dozor*. In particular, BSF has a higher recall rate than both *Dozor* and *DIALS*, which means that it can correctly detect more spots. The precision rate of our model is lower than that of *Dozor*; data-driven artifacts (*e.g.* ice rings) in the images will cause our model to incorrectly classify artifacts spots, leading to a high number of false positives. In terms of the F1 score, our model outperforms both *DIALS* and *Dozor*. Finally, in the image-level comparison, our model has better results than *DIALS* and *Dozor* predictions for all metrics, which shows the robustness of the BSF model. From a user perspective, BSF provides consistent predictions on images with partial or weak reflections.

### 4.4. Ablation study

We also performed in-depth ablation studies to evaluate the effectiveness of the proposed pre- and post-processing strategies (we only report the spot-level metrics). Table 3 ▸ shows the performance using different radii to generate ground truth masks, from which we can observe that both overly large and overly small radii will affect the model performance, leading to lowered performance. We experimentally found that radius = 10 gives the best results. We further verified the effectiveness of the proposed post-processing, including the large-spot splitting (LSS), ice ring spot removal (IRSR) and tilted detector calibration (TDC); the results are shown in Table 4 ▸. We observed that only using LSS does not improve the model performance because, without any further false-positive spot removal, splitting large spots into sub-spots generates many new false-positive spots. The IRSR by itself provides limited improvement, but it can improve the F1 score by a large amount when combined with LSS. Finally, the TDC further increased the F1 score by generating more accurate ice rings for IRSR.

### 4.5. Qualitative examples

**Figure 6:**
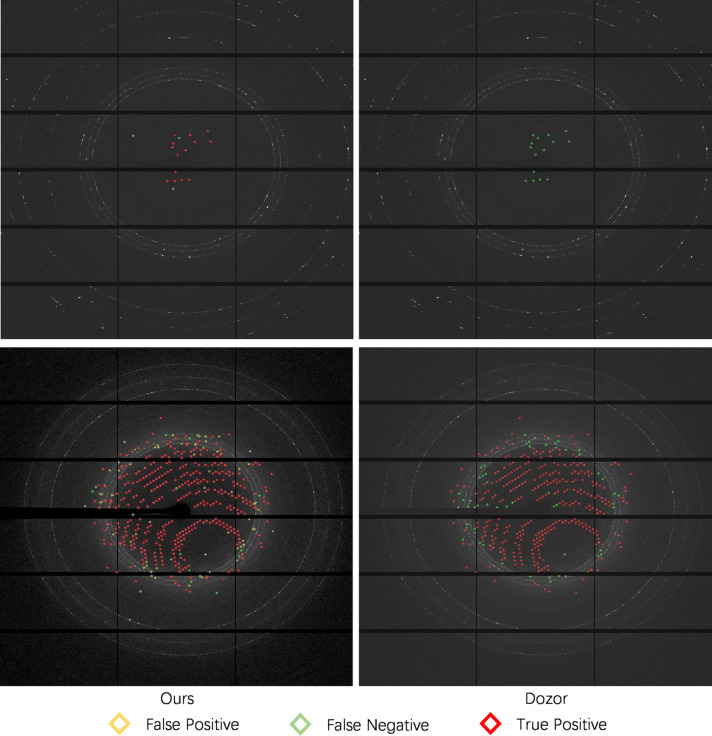
Qualitative examples of BSF (left) and *Dozor* (right). *Dozor* has many more false-negative predictions (in green). Full-resolution figures are included in the supporting information: the top left corner, BSD image bsd_000010_301, processed with BSF in Fig. S1; the top right corner, BSD image bsd_000010_301, processed with *Dozor* in Fig. S2; the bottom left corner, BSD image bsd_000023_18, processed with BSF in Fig. S3; the bottom right corner, BSD image bsd_000023_18, processed with *Dozor* in Fig. S4.

**Figure 7:**
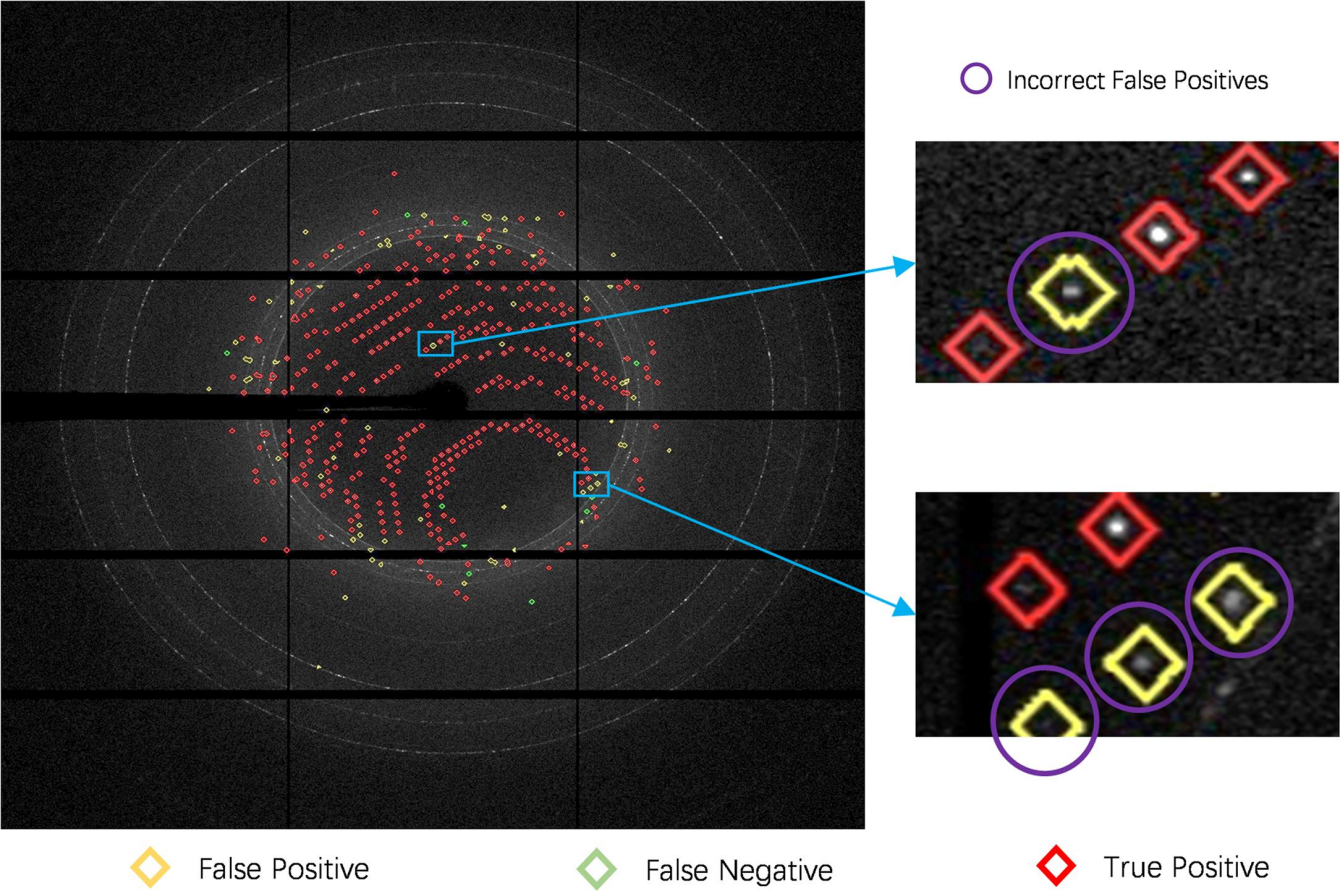
Examples of incorrect false negatives in our predictions. We can observe that four incorrect false-positive spots marked with yellow diamonds surrounded by purple circles should have been marked as true-positive spots; the error is probably due to incorrect labelling.

To illustrate the spot detection quality of the proposed method, we visualized some qualitative examples and compared them with detection results from the *Dozor* application (shown in Fig. 6 ▸). We observed that our model detected harder-to-detect spots and gave fewer false negatives. In particular, *Dozor* cannot detect spots in the top images (images with partial reflections or weak reflections) while our model misses a single spot. In the bottom images, our model gave fewer false negatives and detected more true spots than *Dozor*, though it had more false-positive spots and mis-classified some artifacts as spots. Moreover, we find that some false positives in our predictions may be true positives due to the incorrect human annotation as shown in Fig. 7 ▸. One possible reason could be that we annotate the ground truth spots according to the predictions of the *Dozor* software, so some spots that are not detected by *Dozor* are ignored by us as well. *Dozor* predictions are carefully analyzed by hand; expert crystallographers add and remove reflections as needed, generating the best possible annotated frames, with the caveat that minor errors might be introduced.

### 4.6. Discussion and future work

We observe from Table 2 ▸ that, although our model has a high recall rate and outperforms both software models in terms of precision and F1 score, the improvement is limited when compared with *Dozor*. Moreover, in the bottom part of Fig. 6 ▸, there are still some unresolved false positives generated by our model, which is consistent with the relatively low precision rate in Table 2 ▸. Therefore, there is still room for improvement in our model, where we plan to adopt different deep-learning backbones [*e.g.* DepLab (Chen *et al.*, 2018 ▸) and ResUNet (Zhang *et al.*, 2018 ▸)] and include more expert knowledge into the post-processing processes for false-positive removal. In addition, the speed of our model is four images per second, which cannot keep pace with the real time data collection rate. We plan to use teacher–student frameworks and adopt ‘knowledge distillation’ (Xie *et al.*, 2018 ▸) to improve the performance of fast and light-weight neural networks in our future work.

From the end-user perspective, that of a protein crystallography beamline instrumentation scientist at a synchrotron facility or of a structural biologist requiring access to protein crystallography instrumentation, ignoring good spots lying close to ice rings, though not ideal, is an acceptable solution while performing rastering experiments. In a rastering experiment, users rapidly scan the sample and analyze each frame using the spotfinder. Since a large fraction of the protein crystals, especially when studying challenging projects, diffract to a relatively low resolution of 4 Å or lower and the first ice ring appears at 3.9 ± 0.07 Å, this ultimately results in no penalty. However, for samples diffracting to higher resolution, reflections lining up with the ice rings will be ignored, resulting in under-counting of the true number of reflections. Since rastering is designed to locate the best diffracting regions, any sample penalty for under-counting reflections in ice ring regions is the same for all frames and has the same impact on all images, resulting in unnoticeable differences. In other words, ignoring good reflections in ice ring regions has no impact on the rastering experiments. Note that spot finding and spot predictions used for data integration have other requirements when it comes to reflections in ice rings.

A common workflow followed by many crystallography groups accessing synchrotron facilities is to screen dozens to hundreds of initial crystals to identify conditions yielding protein diffraction, and then perform rounds of optimization of the initial crystallization conditions to yield higher-resolution diffraction suitable for structure solution at the desired or best achievable resolution. The initial step in this workflow relies on screening many potential samples, using rastering with a step size as small as or smaller than the crystal size to search for large-molecule (macro-molecule) diffraction (Bragg spots), a promising sign for potential improvement downstream of the workflow. These promising crystals often yield only a few reflections. Sometimes they diffract well enough for successful indexing but rarely for successful complete data collection and analysis. For many projects, it is key to detect these rare crystals with a few spots sooner so that there is the potential for optimization. Many groups return to steps upstream of this workflow to modify the construct so that they express and purify more stable entities when all crystallization trials appeared to fail. A spotfinder with increased sensitivity will increase the likelihood and speed in obtaining structural information by allowing detection of weak diffraction in the early steps of the workflow.

BSF has the potential to noticeably improve crystal detection and the best diffracting volume from large crystals, especially for crystal samples with weak reflections. To further improve outcomes from rastering experiments, we will use coordinates derived from the BSD dataset output reflections and attempt to index each pattern to potentially improve the BSD results after extending reflection positions from calculated predictions. For these results, indexing and additional reflections will be used to improve heat maps and the likelihood for optimal data collection.

### 4.7. Availability of BSD data and BSF code

The BSD dataset, consisting of 304 diffraction images from rastering experiments, is available on Zenodo (https://doi.org/10.5281/zenodo.10667264); details of the dataset are included in the Zenodo dataset description. BSF code is available at GitHub (https://github.com/DJX1995/BraggSpotFinder).

## 5. Conclusions

In this work, we present a new Bragg spot detection dataset called BSD, which builds on high-resolution images. We provide a detailed data collection process and present the dataset statistics and BSF, a strong baseline AI spotfinder for efficient Bragg spot detection. BSF contains image pre-processing, a U-Net backbone for image segmentation and carefully designed post-processing steps, including LSS using a watershed algorithm, IRSR and TDC. Experiments on the BSD dataset show that BSF gives a better performance than two popular software packages (*Dozor* and *DIALS*), demonstrating that this is an appropriate framework within which to support future extensions and improvements.

Detectors are becoming faster, and beamlines brighter, allowing faster rastering with step sizes as small as 0.5 µm from crystals as small as 0.5 µm. An improved sensitivity spotfinder that can run as fast as new detectors is required. This will truly unleash the full potential that can be achieved in these new facilities. In return, it will speed up potential discoveries relying on macromolecular crystallography. However it will require improvements to keep up with detector speeds. 

## Supplementary Material

Supporting figures. DOI: 10.1107/S1600576724002450/jo5096sup1.pdf

URL: https://doi.org/10.5281/zenodo.10667264

## Figures and Tables

**Table 1:** Dataset statistics

Splits	No. of images	No. of spots
Train	245	52870
Test	59	14766

**Table 2:** Experimental comparisons with *Dozor* and *DIALS* The best results are shown in bold.

	Spot level			Image level		
Methods	Recall	Precision	F1 score	Recall	Precision	F1 score
*Dozor*	0.859	**0.935**	0.895	0.784	0.884	0.822
*DIALS*	0.487	0.759	0.593	0.376	0.782	0.466
BSF	**0.897**	0.904	**0.900**	**0.906**	**0.897**	**0.899**

**Table 3:** Experiment results using different radii to generate segmentation masks

Radius	Recall	Precision	F1 score
4	0.562	0.865	0.714
7	0.885	0.848	0.866
10	0.897	0.904	0.900
13	0.898	0.864	0.881

**Table 4:** Effectiveness of the proposed post-processing

LSS	IRSR	TDC	Recall	Precision	F1 score
No	No	No	0.943	0.798	0.864
Yes	No	No	0.938	0.796	0.861
No	Yes	No	0.886	0.853	0.869
Yes	Yes	No	0.898	0.876	0.887
Yes	Yes	Yes	0.908	0.891	0.899
