# Supplementary material for: Bragg Spot Finder (BSF): a new machine-learning-aided approach to deal with spot finding for rapidly filtering diffraction pattern images
Source: J Appl Crystallogr. 2024 Apr 26;57(Pt 3):670–80. doi: 10.1107/S1600576724002450 (PMC11151665; doi:10.1107/S1600576724002450)
Supplement: Supplementary file 1 [file j-57-00670-sup1.pdf]

JOURNAL OF APPLIED CRYSTALLOGRAPHY

Volume 57 (2024)

Supporting information for article:

Bragg Spot Finder (BSF): a new machine learning aided approach to deal with spot finding for rapidly filtering diffraction pattern images

Jianxiang, Zhaozheng, Dale, Herbert J. and Jean

The qualitative examples in Fig. 6 are very detailed. In order to improve the clarity of that figure, we present each corner of the figure as a full page image, with the top left corner, BSD image bsd_000010_301, processed with BSF in Fig. S1, the top right corner, BSD image bsd_000010_301 processed with Dozor in Fig. S2, bottom left corner, BSD image bsd_000023_18, processed with BSF in Fig. S3, the bottom right corner, BSD image bsd_000023_18 processed with Dozor in Fig. S4.

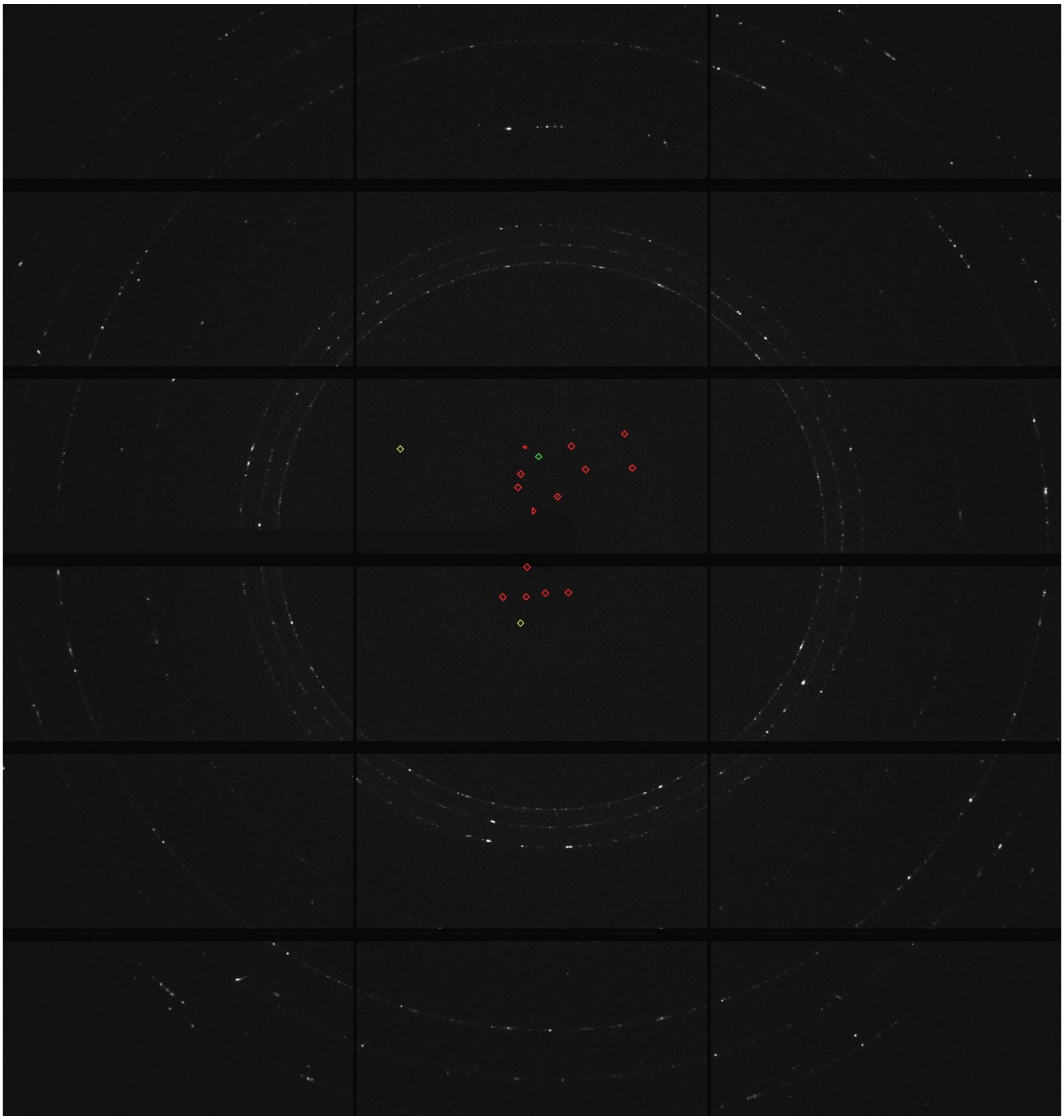

Figure S1 Full resolution image of bsd_000010_301.cbf processed by BSF, top left corner of Fig. 6.

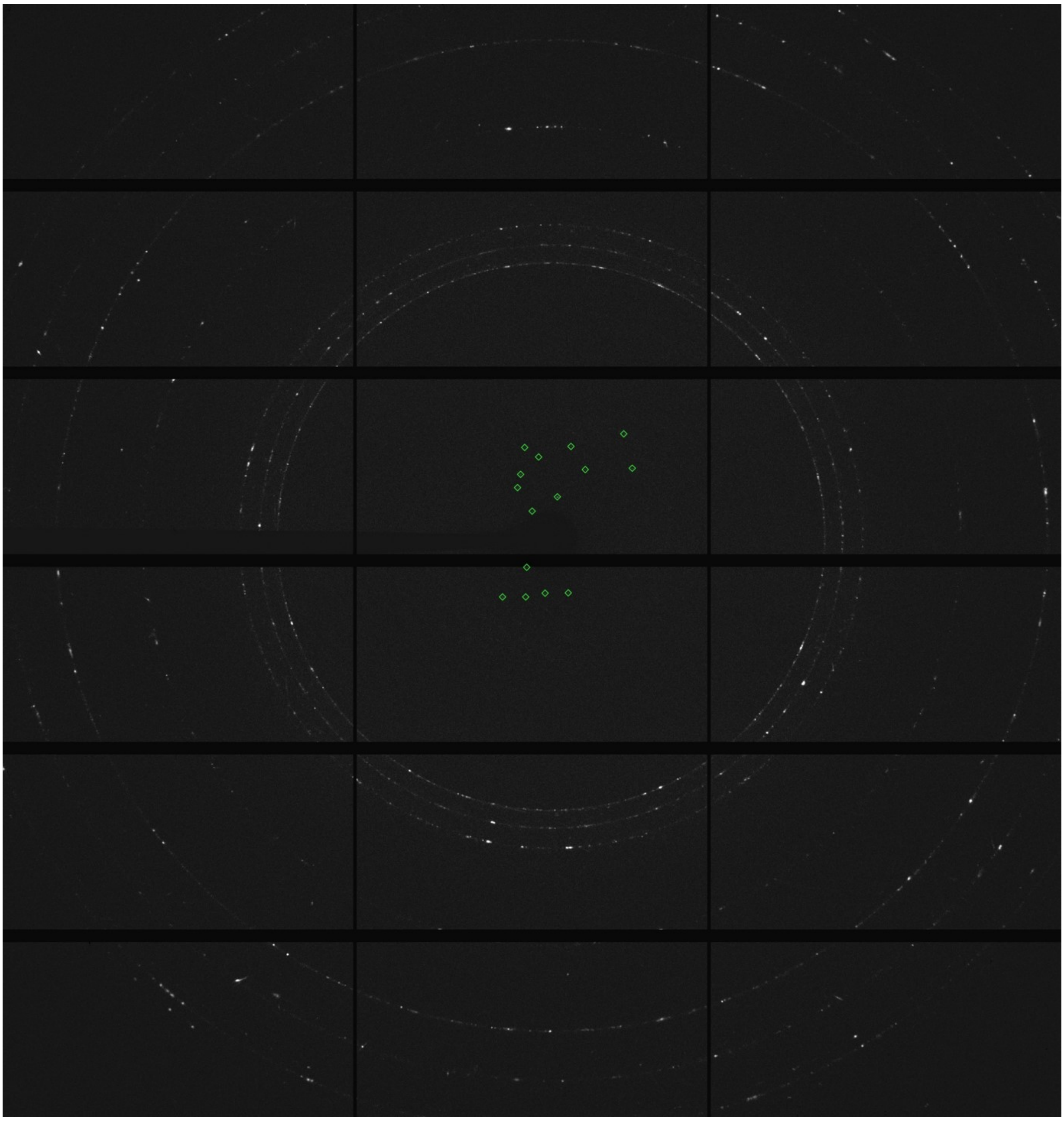

Figure S2 Full resolution image of bsd_000010_301.cbf processed by dozor, top right corner of Fig. 6.

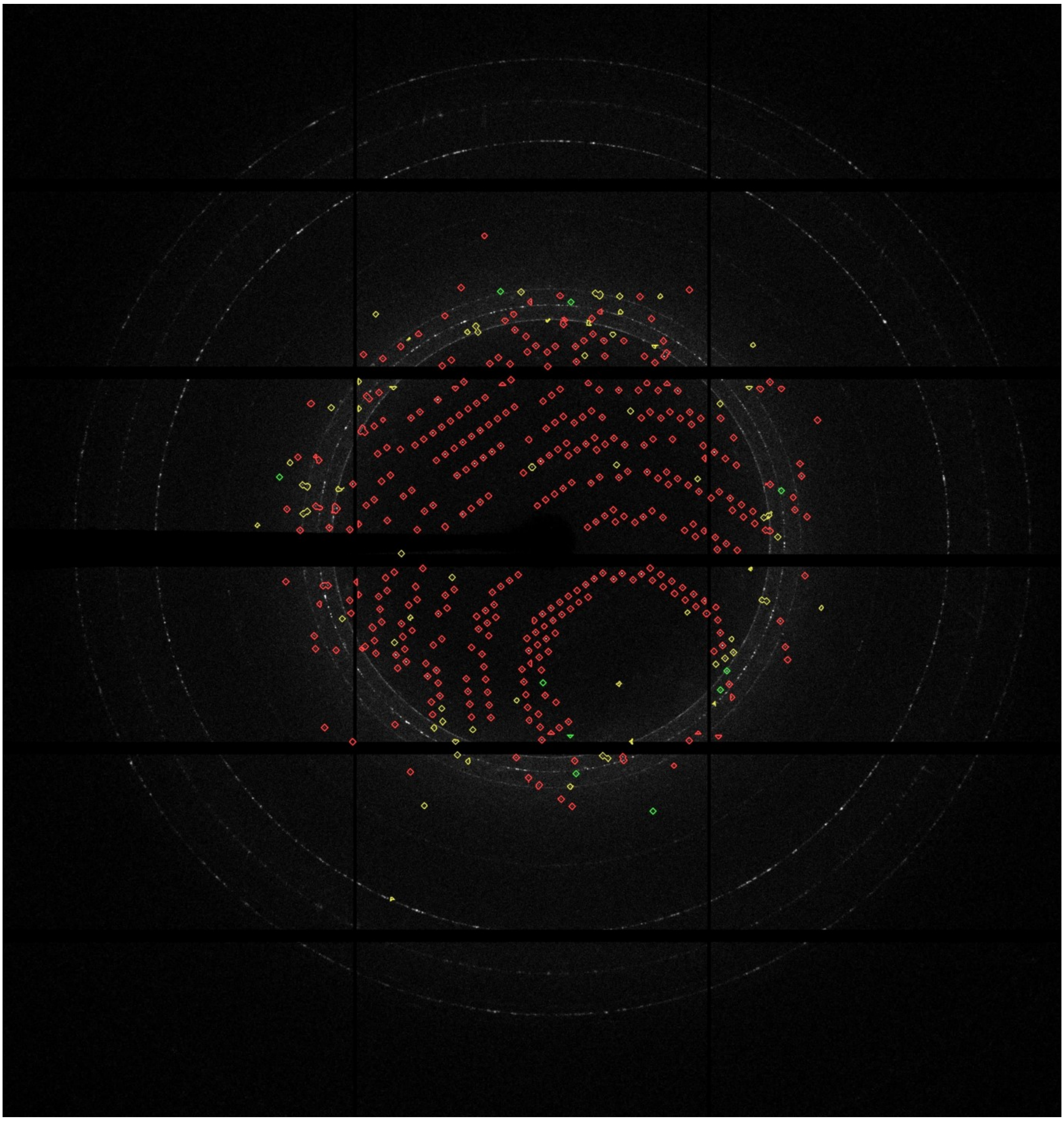

Figure S3 Full resolution image of bsd_000023_18.cbf processed by BSF, bottom left corner of Fig. 6.

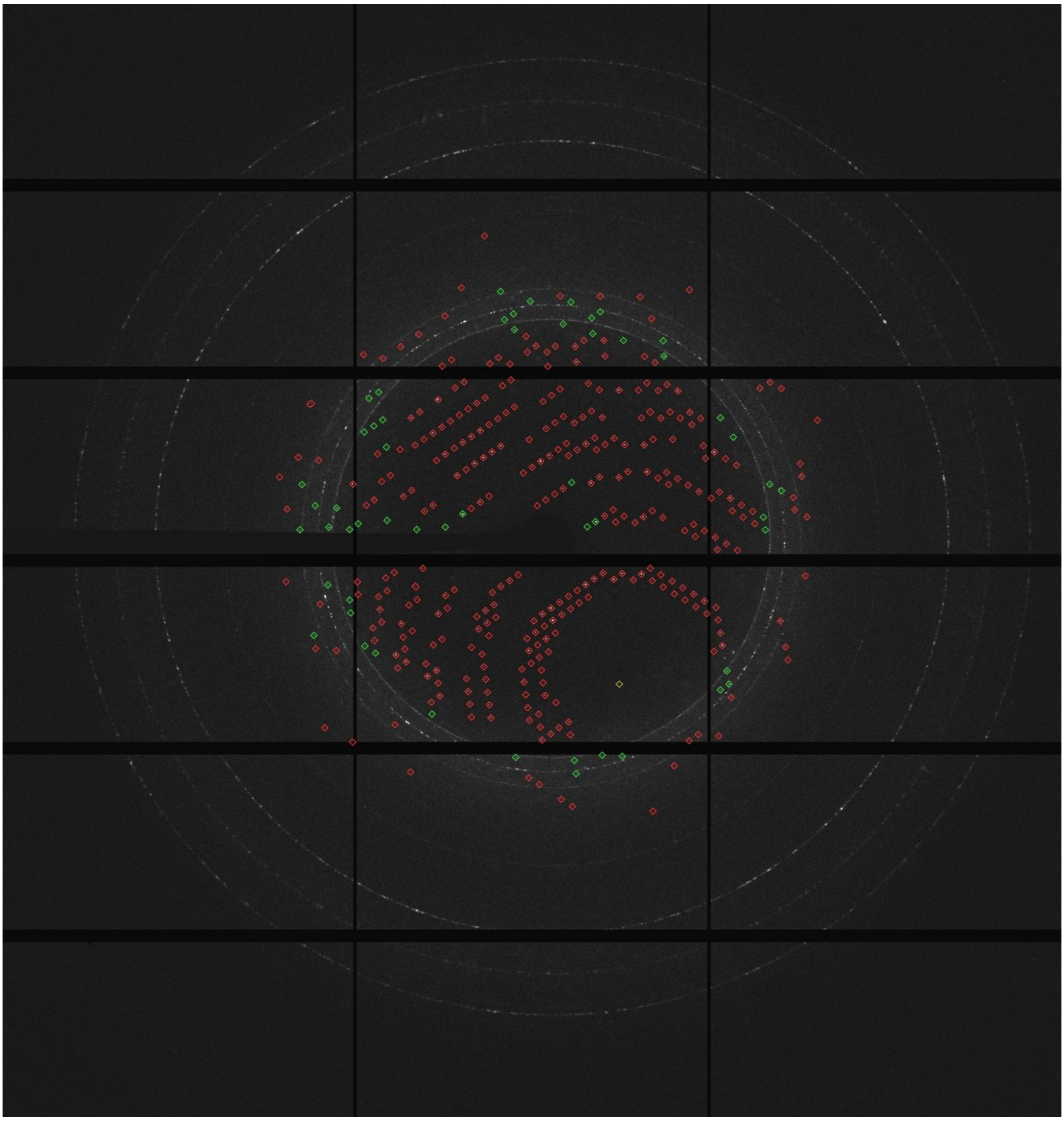

Figure S4 Full resolution image of bsd_000023_18.cbf processed by dozor, bottom right corner of Fig. 6.
